# Stress indicators in past Mesopotamian populations from the Hamrin Basin of the middle Tigris

**DOI:** 10.1537/ase.250921

**Published:** 2025-12-09

**Authors:** Hitoshi Shimakura

**Affiliations:** 1 Laboratory of Physical Anthropology, Division of Biological Sciences, Graduate School of Science, Kyoto University, Kyoto, Sakyo, Kyoto, 606-8502 Japan

**Keywords:** Mesopotamia, paleopathology, stress indicator, chronological differences, Iraq

## Abstract

Bioarchaeological studies of people in Mesopotamia are scarce compared with other regions. This study investigated the prevalences of cribra orbitalia, enamel hypoplasia, and porotic hyperostosis on human skeletal remains excavated from the Hamrin Basin in the middle Tigris River, spanning from ~4000 BC to the Islamic period. The prevalence of cribra orbitalia and enamel hypoplasia was high and remained around 70–80% until the first century. The prevalence of stress indicators decreased in the Islamic period. The hypothesis that the rapid cooling and aridification in West Asia around 2000 BC increased stress levels was not statistically supported in this study. It seems that Mesopotamians were exposed to generally higher stresses than the Egyptians in contemporary periods.

## Introduction

Mesopotamia is one of the earliest civilization centers in the world. The fertile land between the Tigris and Euphrates rivers has been cultivated since around 8500 BC (Moore, 1982; Chase-Dunn et al., 2012). Despite the existence of numerous clay tablets that document administrative information about the society (Ohtsu and Shimogama, 2014), very little is known about the health status of the people who lived in ancient Mesopotamia (Sołtysiak, 2013).

## Paleopathological Studies of Stress Indicators

Observation of paleopathological stress indicators is a useful way to understand the general health status of ancient people (Lallo and Rose, 1979; Goodman et al., 1984; Yamamoto, 1988; Goodman and Rose, 1991). Commonly used stress indicators are cribra orbitalia (CO), porotic hyperostosis (PH), enamel hypoplasia (EH), and Harris lines (Buikstra and Ubelaker, 1994). In the Nile Basin, Starling and Stock (2007) noted that the frequency of EH increased from about 40% to 70% at the advent of agriculture. The authors suggested that increased population density triggered the spreading of infectious diseases and seasonal food shortages, which causes immune deficiency. Hillson (1979) also studied the chronological changes in the frequency of EH in populations along the Nile. He found that the frequency of EH was about 70% in Upper Egypt (Badari) in 5000–3000 BC and about 60% in Central Egypt (Sidmant) in 2000 BC. Then, the frequency dropped to about 40% in Nubia (Kerma) in 1720–1550 BC. Hillson (1979) attributed the high frequency of EH in Badari and Sidmant to excessive clothing, avoidance of exposing children to sunlight, vitamin deficiency due to breast milk from undernourished mothers, and the long lactation period (3–4 years). He also pointed to febrile illnesses caused by depressed immunity resulting from nutritional and vitamin deficiencies.

The impact of stress on human health has also been studied in Iran in the east of Mesopotamia. Two sites, namely Gohar Tepe (c. 2500 BC: Sołtysiak and Mahfroozi, 2008) and Dinka Tepe (1400–450 BC: Wang, 2012), situated in Hassanlu, northwest Iran, have been found to have a frequency of EH of around 70%. It was hypothesized that population concentration and climatic changes may have led to nutritional deficiencies, resulting in a higher frequency of EH.

The phenomenon of increased stress indicators during the shift from hunting and gathering to agriculture is well documented in North America (Sciulli, 1978; Cook and Buikstra, 1979; Goodman et al., 1980; Larsen, 2006). A prehistoric population from Illinois (Goodman et al., 1980) showed an increase in EH frequency with the degree of dependence on agriculture (hunter-gatherer period, 45%; agricultural transitional period, 60%; agricultural period, 80%). The research indicated that the increase in frequency was due to a nutritional deficiency in maize agriculture, leading to a decline in immunity, as well as to the spread of infectious diseases caused by the high population density.

## Studies on Stress Indicators in Mesopotamia

There are surprisingly fewer studies of this kind in Mesopotamia. The only study that examined chronological changes in the frequency of stress indicators in Mesopotamia was Tomczyk et al. (2007), who analyzed 132 human skeletal remains from three sites in the Middle Euphrates River Basin covering five periods (2100–1700 BC, 1500–600 BC, 200–650 AD, 650–1200 AD, 1850–1950 AD), and reported the prevalence of EH in each period. The mean frequency of occurrence by tooth type (all tooth types except third molars) was 16.3% in the Shakanaku/Old Babylonian period (2100–1700 BC), 26% in the Late Roman/Byzantine period (200 AD–650 AD), and 15.7% in the Early Islamic period (650–1200 AD). However, the frequency of EH occurrence varies by tooth type (Goodman and Armelagos, 1985; Goodman and Rose, 1990). These values cannot be compared directly with most other data which was documented per individual.

There are two case reports on Mesopotamian populations. Mckenzie (1999) conducted a paleopathological study of adult bones from Tell Leilan, Syria (2300–2000 BC), northern Mesopotamia, and observed CO in four out of six individuals (67%). Bertoldi et al. (2014) investigated the presence of EH in 13 individuals excavated from Tell Beydar, northern Syria (2550–2350 BC), and found EH in 9 individuals (69.2%).

West Asia became colder and drier around 2200 BC and this climate abnormality continued for 300 years (Weiss et al., 1993; Bar-Matthews et al., 1997; Lemcke and Sturm, 1997; Cullen et al., 2000; Weiss, 2000; Zanchetta et al., 2014; Watanabe et al., 2019). This climate change (the 4.2 ka BP event) brought severe cold and aridity to West Asia such as a 3–4°C degree drop in sea surface temperatures and a reduced rainfall due to the weakening of the monsoon (Kajita et al., 2018). Weiss et al. (1993) suggested that Tell Leilan was abandoned due to windblown sand caused by this climatic change. Mckenzie (1999) also attributed this climatic change to one of the possible causes of the suppressed health status of the people of Tell Leilan.

During the Islamic period, this region entered a period of social stability and cultural development known as the ‘Islamic Golden Age’ (c. 750–1258 AD) (Lapidus, 1992; Falagas et al., 2006; Renima et al., 2016). Tomczyk et al. (2007) suggested that the quality of life in the Islamic period was relatively better due to economic prosperity. However, the prevalence of stress indicators has not been fully investigated.

This study examined the prevalence of stress indicators observed in human skeletal remains excavated from the Hamrin Basin, located in the middle Tigris region, spanning roughly from 5000 BC to the Islamic era, to understand the change in health status among people in Mesopotamia. The findings are compared with data gathered from Iran and Egypt and with the results of a study conducted by Tomczyk et al. (2007).

## Geography

**Figure 1.**
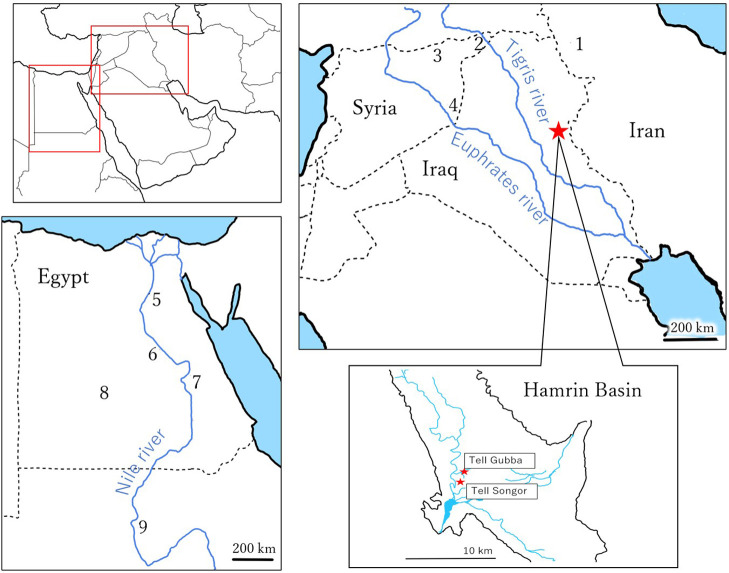
Map of archaeological sites in the Mesopotamia and other regions referred to in the text. The location of the Hamrin Basin is indicated by a star. (1) Gohar Tepe/Dhinka Tepe, (2) Tell Leilan, (3) Tell Beydar, (4) Tell Ashara, (5) Sidmant, (6) Badari, (7) Nakada, (8) Dakhula, (9) Kerma.

The Hamrin or Himrin (this study uses the former following the excavation report ‘Al Rafidan II’ (Fujii, H. (1981)) Basin, including Tell Gubba and Tell Songor, is located around 130 km northeast of Baghdad. The Diyala River, a tributary of the Tigris, flows through this region (Figure 1).

Annual precipitation is approximately 300 mm, making the Hamrin Basin a marginal region for rain-fed agriculture. Irrigated agriculture was also practiced in the lowlands, with wheat and barley cultivated as the primary crops (Al Rafidan II). More than 70 relics (tells) of various sizes are scattered throughout the area. Important trade routes such as the Great Khorasan Road pass through this region, connecting Baghdad to the other regions (see Fujii, 1981; Gibson, 1982).

According to Renette (2009), the Hamrin Basin in the 3rd millennium BCE lacked extensive agricultural plains, but long irrigation canals were already in existence. Considering the similarities in environment, topography, and ethnographic records from Luristan in western Iran, the primary subsistence activity was likely pastoral nomadism, with small-scale but localized agriculture also being practiced. The discovery of Hamrin Basin-specific pottery known as Scarlet Ware, along with architectural styles, burial practices, and metal artifacts, suggests that the Hamrin Basin underwent a unique cultural development distinct from central and southern Mesopotamia. It is instead considered to have been more culturally aligned with sites in southwestern Iran and northern Iraq (Renette, 2009; Del Bravo, 2014; Oselini, 2018).

## Materials and Methods

H. Fujii and his colleagues at the Institute for Cultural Studies of Ancient Iraq, Kokushikan University conducted excavations at Tell Gubba and Tell Songor in this area from 1977 to 1980. These sites contained layers dating from the Ubaid period to the Islamic period and yielded more than 300 graves. No previous studies have explicitly addressed the social status of the deceased. Although burial practices within the same period appear to be uniform, differences in the presence of grave goods (pots, seals, animal skulls, and other pottery) have been noted. From the Ubaid layers at Tell Songor, numerous artifacts such as awls, sickle blades, and scrapers have been recovered, whereas items identifiable as stone arrowheads are scarcely found. From this evidence, it has been suggested that by the Ubaid period subsistence was no longer centered on hunting but had already shifted toward agriculture (Al Rafidan II). The Hamrin skeletal remains from these two Tells are housed in the Kyoto University Museum. This material comprises populations from different chronological periods excavated from the Hamrin Basin, making it a valuable source for observing diachronic changes in health status within a specific locality. Investigating these changes and their underlying factors may provide important insights into the lifeways of Mesopotamian people, which remain largely unknown. The author examined human skeletal remains from the Ubaid period (c. 5000–4000 BC), the Jemdet Nasr period (3100–2900 BC), the Isin Larsa/Old Babylonian period (c. 2000–1500 BC), the Neo-Assyrian period (c. 900–600 BC), the Parthian period (c. 250 BC–220 AD), and the Islamic period (c. 630 AD–) (Table 1).

The Hamrin Basin people are characterized by a relatively dolichocephalic (long-headed) crania and are believed to have remained largely unchanged until the Parthian period, as in southern Mesopotamia. However, at some point during the Parthian period, the arrival of brachycephalic (short-headed) groups likely introduced morphological diversification in cranial traits (Ogihara et al., 2009). The chronology of each period follows Ogihara et al. (2009).

This study examined the occurrence of CO (*N* = 211), PH (*N* = 152), and EH (*N* = 118), following Buikstra and Ubelaker (1994) for CO and PH, and Yamamoto (1988) for EH. Incisors and canines were used for the EH observation because the mandibular canines are the most useful due to their longer crown formation period (Goodman et al., 1980; Goodman and Armelagos, 1985; Yamamoto, 1988; Goodman and Rose, 1990), and the incisors and canines are better preserved in samples. Because EH is usually formed after 3.5 years of age (Goodman et al., 1984; Corruccini et al., 1985; Yamamoto, 1988), individuals estimated to be older than 3.5 years were selected. The frequency of occurrence of EH was evaluated on an individual basis following most of the previous studies. However, to compare the results with the work by Tomczyk et al. (2007), frequency in tooth type was also investigated. Following Tomczyk et al. (2007), when both side teeth were preserved in the same individual, they were counted independently.

Henriquez and Oxenham (2017, 2020) recommended an objective and quantitative method to identify and analyze EH, i.e. using a confocal microscope, which is also useful for surveying individual life histories. However, this study used a conventional field method following the ‘Data Collection Codebook’ for the Global History of Health Project (Steckel et al., 2006; https://economics.osu.edu/global-history-health-project).

The severity of each stress indicator was also recorded. Following Buikstra and Ubelaker (1994), microporosity changes were classified as score 1, clear porosity changes as score 2, and large pore-to-pore changes as score 3 for CO and PH. Regarding EH, a linear shallow groove(s) across the labial or buccal side of the crown was designated as score 1, followed by a pit-like defect as score 2, and a deep sulcus that sometimes reaches the dentin was designated as score 3.

Sex was not considered because there were insufficient skeletal remains to analyze differences by sex. Fisher’s exact test was used to test for significant differences in the frequency of occurrence by period, and the Bonferroni method was used for multiple comparisons. The method of estimating age followed Buikstra and Ubelaker (1994) (Table 2).

## Results

Table 3a summarizes the frequency of occurrence of the three stress indicators. Concerning CO, the highest frequency was 88.2% in the Ubaid period. The frequency was slightly lower in later periods: Jemdet Nasr, 77.8%; Isin Larsa/Old Babylonian, 64.7%; Neo-Assyrian, 76.2%. The Islamic period had the lowest frequency (47.4%). Multiple comparisons using Fisher’s exact test showed a significant difference only between the Ubaid and Islamic periods (*P* = 0.0228). However, the Ubaid period was excluded, as only infant and child samples were available. Multiple comparisons revealed no significant difference (*P* > 0.95).

Table 3b shows the difference in the frequency of CO between late childhood or younger and subadult or older. In the Islamic period, where the sample size is sufficient, CO occurrence was significantly more frequent in the late childhood or younger group than in the late adult or older group (*P* = 0.00043).

The frequency of PH was obtained from only three periods. It was 25% in the Ubaid period, 7.1% in the Isin Larsa/Old Babylonian, and 9.5% in the Islamic period. Except for the Islamic period, the sample size was too small and no statistical analysis was possible.

The frequency of EH was highest in the Isin Larsa/Old Babylonian periods (85.7%) and was lowest in the Islamic period (52.4%). No data were available for the Ubaid period. Due to sample size limitations in conducting multiple comparisons, a pairwise comparison was performed between the Isin Larsa/Old Babylonian period and the Islamic period. The result was *P* = 0.021 (*P* < 0.05), indicating a statistically significant difference.

The EH occurrence frequency by tooth type is shown in Table 4a. The frequency of EH occurrence was variable by tooth type. The frequency was generally higher in the mandibular canine than in other tooth types. The frequency of EH in the mandibular canines follows the same trend as that calculated per individual (Table 3). It is the lowest in the Islamic period (47.6%).

Table 5 shows the frequencies of individuals who show severe symptoms (scores 2 or 3). Regarding CO, this frequency varies greatly between periods (from 5.9% to 42.9%). However, no statistically significant difference was observed. Likewise, only a small number of individuals were scored as 2 or 3 regarding PH and EH.

## Discussion

This study aimed to investigate temporal changes in the frequency of three stress indicators in populations residing in the Tigris region. Tomczyk et al. (2007) focused on populations along the Euphrates River (Tell Ashara) in eastern Syria. Despite the two study areas being 460 km apart, this study greatly supplemented their data (Table 4b) because they reported the frequencies of EH on a tooth type basis (incisor and canine), not per individual as commonly employed. In their study sample, the Shakanaku/Old Babylonian period (2100–1700 BC) roughly corresponds to the Isin Larsa/Old Babylonian periods in this study chronologically. The EH frequency in the Shakanaku/Old Babylonian period is 54% in the mandibular canine, much lower than the value in the Isin Larsa/Old Babylonian periods (88.2%). The frequency of the Late Roman/Byzantine periods (200–650 AD) is 43%, lower than that in the Parthian period (250 BC–220 AD: 66.7%) and similar to the Islamic period (630AD–: 47.6%). Because Tomczyk et al. (2007) did not show the sample size for each tooth type, a statistical test is impossible. However, the populations along the Euphrates might have experienced stresses less severely compared with those along the Tigris around 2000 BC.

During the Ubaid period, agriculture and nomadic pastoralism were practiced, and irrigation for agriculture was introduced (Oates, 1993; Pollock, 1999). Only CO (88.2%) and PH (25%) data were available from the Ubaid period. Because only non-adult skeletal remains were available for analysis among the samples examined, comparisons with other periods were not appropriate. The causes of CO and PH are diverse: parasitic infections, iron deficiency anemia, folate deficiency, and megaloblastic anemia (Buikstra and Ubelaker, 1994; Fairgrieve and Molto, 2000). PH is also caused by a deficiency of vitamin B12 (Walker et al., 2009). It is impossible to conclude what is responsible for the appearance of CO and PH during the Ubaid period. Future analysis of archaeological artifacts may help to specify the main causes.

The present study did not find significant differences in CO frequencies during the Jemdet Nasr and Isin Larsa/Old Babylonian periods (70–80%). The climate change known as the 4.2 ka BP event, which had a profound impact on various civilizations, including Mesopotamia, Egypt, and the Indus Valley Civilization, has been documented between the Jemdet Nasr and Isin Larsa/Old Babylonian periods (Weiss et al., 1993; Bar-Matthews et al., 1997; Lemcke and Sturm, 1997; Cullen et al., 2000; Weiss, 2000; Staubwasser and Weiss, 2006; Zanchetta et al., 2014; Welc and Marks, 2014; Watanabe et al., 2019). It has been suggested that a drought caused by a reduction in precipitation of approximately 20–30% between BC 2200 and BC 1900 may have led to famine (Staubwasser and Weiss, 2006; Watanabe et al., 2019). When considering only the frequencies of CO and EH during the Isin Larsa/Old Babylonian period, it indeed appears to align with the scenario in which famine occurred as a result of this arid and cold climate, leading to deteriorations in people’s nutritional and health conditions. Furthermore, the emergence of measles in Mesopotamia around BC 3000 has been suggested (Cliff et al., 1993). Given that the Hamrin Basin was located along trade routes, it is also plausible that under conditions of malnutrition, the risk of infection from diseases introduced from other regions might have been considerably elevated.

During the Neo-Assyrian period, the expansion of the national territory and forced migration from conquered areas to urban areas occurred simultaneously. This led to a rapid increase in the population density in urban areas (Parpola, 2004; Wilkinson et al., 2005), which may have led to a deterioration of sanitation and to food shortages. However, no significant increase in frequencies of stress indicators was observed in the Hamrin Basin during the Neo-Assyrian period. Ogihara et al. (2009) also noted that morphological diversification had not yet occurred during this period, suggesting that, at least in the Hamrin Basin, there was no substantial migration from other regions, and it is unlikely that people’s health or living conditions deteriorated significantly.

The frequency of both CO and EH decreased during the Islamic period. The frequency of EH in the Islamic period is similar to that reported by Goodman et al. (1980) for the hunter-gatherer society (45%). Tomczyk et al. (2007) suggested that the development of trading between northern and southern Mesopotamia in the Islamic period enhanced the economy, and hence improved the quality of life. This might have happened for the Hamrin Basin too.

## Neighborhood Comparison

Comparisons between Mesopotamia and neighboring regions are discussed below. In a population belonging to the Badari cultural period in Egypt (c. 4400–4000 BC), the frequency of PH was 79.6% (*N* = 54) (Keita, 2003). This value is significantly higher (*P* < 0.005) than during this study’s Ubaid period (5000–4000 BC; Table 3).

According to some authors, the Badari cultural period is supported by a semi-sedentary and foraging economy or initial agriculture (Keita, 2003). During the Ubaid period, agriculture and nomadic pastoralism were practiced, and irrigation for agriculture was introduced. Changes in subsistence ecology may explain this difference.

**Figure 2.**
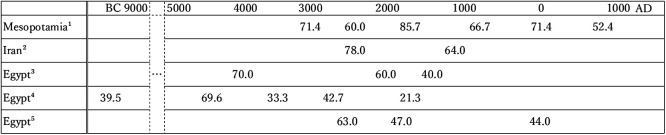
Prevalence of EH in human remains from Mesopotamia, Iran, and Egypt (%). ^1^From this study except for data for c. 3000–2000 BC, which was adopted from Bertoldi et al. (2014). ^2^Data for c. 3000–2000 BC (from Sołtysiak and Mahfroozi, 2008) and 1500–500 BC (from Wang, 2012). ^3^From Hilson (1979). ^4^From Starling and Stock (2007). ^5^From Lovell and Whyte (1999).

In Mesopotamia, the frequency of stress indicators remained high from the early agricultural period and did not decline until the Islamic period. This trend is compared with neighboring populations (Figure 2).

In Iran, the frequency of EH has been investigated in skeletal remains from Gohar Tepe (c. 2500 BC) (Sołtysiak and Mahfroozi, 2008), and the Hassanlu and Dinkha Tepe sites (1400–450 BC) (Wang, 2012) in the Solduz. The age of Gohar Tepe is between the Jemdet Nasr and Isin Larsa/Old Babylonian periods. The age of the Hassanlu/Dinkha Tepe sites partially overlaps the Parthian period. The frequency of EH is 78% (*N* = 19) and is not significantly different from the present study. CO was observed in 40% (*N* = 15) of the individuals, which is not significantly different from the Jemdet Nasr and Isin Larsa/Old Babylonian periods. The overall frequency of EH in human skeletal remains from the Hassanlu/Dinkha Tepe site is 64% (Wang, 2012). This score is similar to the results of this study. A similar value (69.2%, *N* = 13) is reported from Tell Beydar in northern Mesopotamia (2550–2350 BC; Bertoldi et al., 2014). In summary, people experienced high stress, and an EH frequency of around 70% was common between 2500 and 2000 BC in northern Mesopotamia, the Hamrin Basin, and Iran.

More research has been conducted in the Nile Basin (Upper and Lower Egypt and Nubia). According to Starling and Stock (2007), the frequency of EH in the five Nile Basin populations (*N* = 242) is 39.5% (*N* = 38) in Jebel during the hunter-gatherer period and has ranged between 70% and 30% since the beginning of farming. A low value of about 20% was observed in Kerma (2100–1500 BC). Specific reasons, such as food stockpiling through trade, are suggested for this particularly low rate (Starling and Stock, 2007). Hillson (1979) obtained 70–40% frequency rates from the onset of agriculture to 1500 BC. Lovell and Whyte (1999) reported the EH frequencies in middle-class populations from Lower Egyptian Mendesian Dynasty I (3000–2180 BC), the First Intermediate Period (2180–2040 BC), and the Greco-Roman Period (332 BC–395 AD). EH prevalence rate ranges from 63% to 44%. Overall, the frequency of EH in the Nile Basin is lower than that in comparable periods in Mesopotamia, pointing to higher stress levels in Mesopotamia.

## Conclusion

This study revealed that the frequency of stress indicators in the Hamrin Basin population peaked around 2000 BC during the Isin Larsa/Old Babylonian period and remained relatively high until the Islamic period. This trend aligns with findings from southwestern Iran and northern Iraq, where cultural similarities have been observed. While numerous paleoclimatic studies have pointed out that the 4.2 ka BP event had catastrophic impacts on various civilizations, the present study did not find significant evidence for such effects. At the same time, it is reasonable to assume that the climatic deterioration—characterized by increased aridity and cooling—could have facilitated the spread of infectious diseases, and thus its influence cannot be excluded as one of the factors contributing to the peak in the frequency of stress indicators around 2000 BC. These findings highlight the importance of considering multiple environmental and social factors, rather than attributing health stress solely to the 4.2 ka BP event.

## Figures and Tables

**Table 1.** Number of observed individuals.

Period	Calendar year	N
Ubaid	5000–4000 BC	20
Jemdet Nasr	3100–2900 BC	15
Isin Larsa/Old Babylonia	2000–1500 BC	26
Neo-Assyria	900–600 BC	25
Parthia	250 BC–220 AD	14
Islam	630 AD–	162
Total	—	262

**Table 2.** Age distribution (*N*)

	Infant ~3 years	Early childhood 4–6 years	Late childhood 7–12 years	Subadult 13–20 years	Adult >21 years
Ubaid	15	5	0	0	0
Jemdet Nasr	1	3	4	0	6
Isin Larsa/Old Babylonia	0	5	0	6	14
Neo-Assyria	1	1	0	3	10
Parthia	2	1	1	1	8
Islam	32	26	9	32	59

**Table 3a.** Prevalence of stress indicators in the Hamrin Basin (%)

Period	CO	PH	EH
Ubaid	88.2 (15/17)^a^	25.0 (2/8)	—
Jemdet Nasr	77.8 (7/9)	0 (0/6)	71.4 (5/7)
Isin Larsa/Old Babylonia	64.7 (11/17)	7.1 (1/14)	85.7 (12/14)
Neo Assyria	76.2 (16/21)	0 (0/10)	66.7 (4/6)
Parthia	60.0 (6/10)	0 (0/9)	71.7 (5/7)
Islam	47.4 (65/137)	9.5 (10/105)	52.4 (44/84)

^a^ Number of individuals exhibiting symptoms/total number of observed individuals.

**Table 3b.** Prevalence of CO in two age groups (%)

	Late childhood		Subadult
Ubaid	88.2 (15/17)		0 (0/0)
Jemdet Nasr	33.3 (1/3)		100 (5/5)
Isin Larsa/Old Babylonia	100 (1/1)		66.7 (10/15)
Neo Assyria	100 (1/1)		80.0 (8/10)
Parthia	0 (0/1)		62.5 (5/8)
Islam	66.7 (38/57)		35.0 (27/77)

**Table 4.** Prevalence of EH on the frontal teeth (%)

(a) This study						
This study	I^1^	I^2^	C’	I_1_	I_2_	C
Jemdet Nasr (*N* = 7)^a^	0 (0/2)^b^	100 (3/3)	80.0 (4/5)	57.1 (4/7)	60.0 (3/5)	80.0 (4/5)
Isin Larsa/Old Babylonia (*N* = 14)	44.4 (4/9)	9.0 (1/11)	77.8 (14/18)	11.1 (1/9)	25.0 (3/12)	88.2 (15/17)
Neo-Assyria (*N* = 6)	66.7 (2/3)	100 (2/2)	66.7 (2/3)	0 (0/3)	0 (0/3)	57.1 (4/7)
Parthia (*N* = 7)	50.0 (2/4)	0 (0/4)	50.0 (2/4)	0 (0/5)	14.2 (1/7)	66.7 (6/9)
Islam (*N* = 84)	28.7 (29/101)	19.4 (20/103)	23.7 (28/118)	20.7 (24/116)	25.5 (27/106)	47.6 (60/126)

(b) Tomczyk et al. (2007)^c^						
	I^1^	I^2^	C’	I_1_	I_2_	C
Shakanak–Old Babylonia (N = 28)	8	12	43	18	8	54
Late Roman–Byzantine (*N* = 16)	40	18	38	23	27	43
Early Islam (*N* = 50)	11	22	55	0	10	50

(b) Tomczyk et al. (2007)^c^ ^a^ Number of observed individuals. ^b^ Number of the teeth with EH/number of observed teeth. ^c^ Case number for each tooth type is not available.

**Table 5.** The percentages of individuals with severe symptoms (score 2 or 3)

Period	CO	PH	EH
Ubaid	23.5 (4/17)^1^	12.5 (1/8)	—
Jemdet Nasr	22.2 (2/9)	—	0 (0/7)
Isin Larsa/Old Babylonia	5.9 (1/17)	0 (0/14)	0 (0/14)
Neo-Assyria	42.9 (9/21)	—	0 (0/6)
Parthia	20 (2/10)	—	0 (0/7)
Islam	19.0 (26/137)	6.7 (7/105)	2.4 (2/84)

^1^ Number of individuals with a score 2 or 3/the total number of observed individuals.
